# *Hanseniaspora uvarum* from Winemaking Environments Show Spatial and Temporal Genetic Clustering

**DOI:** 10.3389/fmicb.2015.01569

**Published:** 2016-01-20

**Authors:** Warren Albertin, Mathabatha E. Setati, Cécile Miot-Sertier, Talitha T. Mostert, Benoit Colonna-Ceccaldi, Joana Coulon, Patrick Girard, Virginie Moine, Myriam Pillet, Franck Salin, Marina Bely, Benoit Divol, Isabelle Masneuf-Pomarede

**Affiliations:** ^1^Unité de recherche Œnologie, Institut de la Science de la Vigne et du Vin, University BordeauxVillenave d'Ornon, France; ^2^ENSCBP, Bordeaux INPPessac, France; ^3^Department of Viticulture and Oenology, Institute for Wine Biotechnology, Stellenbosch UniversityMatieland, South Africa; ^4^Institut National de la Recherche Agronomique, Institut de la Science de la Vigne et du Vin, USC 1366 Institut National de la Recherche AgronomiqueVillenave d'Ornon, France; ^5^Centre de Recherche Pernod RicardCréteil, France; ^6^BiolaffortBordeaux, France; ^7^Institut National de la Recherche Agronomique, UMR Biodiversité Gènes et Ecosystèmes, PlateForme GénomiqueCestas, France; ^8^Bordeaux Sciences AgroGradignan, France

**Keywords:** *Hanseniaspora uvarum*, wine, intraspecific diversity, microsatellites, phenotypic screening

## Abstract

*Hanseniaspora uvarum* is one of the most abundant yeast species found on grapes and in grape must, at least before the onset of alcoholic fermentation (AF) which is usually performed by *Saccharomyces* species. The aim of this study was to characterize the genetic and phenotypic variability within the *H. uvarum* species. One hundred and fifteen strains isolated from winemaking environments in different geographical origins were analyzed using 11 microsatellite markers and a subset of 47 strains were analyzed by AFLP. *H. uvarum* isolates clustered mainly on the basis of their geographical localization as revealed by microsatellites. In addition, a strong clustering based on year of isolation was evidenced, indicating that the genetic diversity of *H. uvarum* isolates was related to both spatial and temporal variations. Conversely, clustering analysis based on AFLP data provided a different picture with groups showing no particular characteristics, but provided higher strain discrimination. This result indicated that AFLP approaches are inadequate to establish the genetic relationship between individuals, but allowed good strain discrimination. At the phenotypic level, several extracellular enzymatic activities of enological relevance (pectinase, chitinase, protease, β-glucosidase) were measured but showed low diversity. The impact of environmental factors of enological interest (temperature, anaerobia, and copper addition) on growth was also assessed and showed poor variation. Altogether, this work provided both new analytical tool (microsatellites) and new insights into the genetic and phenotypic diversity of *H. uvarum*, a yeast species that has previously been identified as a potential candidate for co-inoculation in grape must, but whose intraspecific variability had never been fully assessed.

## Introduction

*Hanseniaspora uvarum* (anamorph *Kloeckera apiculata*) is an apiculate yeast species frequently found on mature fruits (Spencer et al., 1992; Morais et al., 1995) and particularly on grapes where it forms part of the grape and fermentation microbiome.Its association with grapes and the first stages of alcoholic fermentation (AF) has been reported repeatedly during the last century (Castelli, 1955; Schütz and Gafner, 1993; Hierro et al., 2006) and for most—if not all—vineyard regions worldwide (Heard and Fleet, 1985; Holloway et al., 1990; Mateo et al., 1991; Comi et al., 2001; Beltran et al., 2002; Jolly et al., 2003; Combina et al., 2005; Li et al., 2010; Zott et al., 2010; Kachalkin et al., 2015). *H. uvarum* is also frequently isolated from other fermented beverages such as cider (Lachance, 1995; Cabranes et al., 1997; Valles et al., 2007; Pando Bedrinana et al., 2012), palm wine and cashew juice (Owuama and Saunders, 1990), tequila (Bilbao et al., 1997), sugar-cane aguardente (Morais et al., 1997), etc. It is part of the natural microbiome of many fermented food processes, including coffee (Masoud et al., 2004) and cocoa (Batista et al., 2015) fermentations. In some biotechnological processes such as yogurt (Kosse et al., 1997), orange juice (Renard et al., 2008), beer (Wiles, 1950), and honey (Pulvirenti et al., 2009) production, *H. uvarum* is considered as a spoilage species. *H. uvarum* also displays industrially relevant antagonistic properties against the development of molds responsible for fruit spoilage. The species is thus extensively assessed as a biocontrol agent against *Botrytis cinerea* (gray mold) on grapes and strawberries (Long et al., 2005; Liu et al., 2010a,b; Cai et al., 2015), *Penicilium* spp. (fruit rot) on citrus (Long et al., 2005), *Colletotrichum capsici* (fruit rot) on chili (Basha and Ramanujam, 2014), etc., while the underlying mechanisms of action are actively studied (Liu et al., 2014; Pu et al., 2014). The ecological extent of *H. uvarum* is large: it has been collected from soils (Capriotti, 1955), plants (Sláviková et al., 2009), insects (Nguyen et al., 2007), birds (Kocan and Hasenclever, 1972), molluscs (de Araujo et al., 1995), and shrimps (Pagnocca et al., 1989), while its occurrence as clinical isolate on humans is rare and considered as opportunistic (Emmanouil-Nikoloussi et al., 1994; Garcia-Martos et al., 1999).

In winemaking, the presence of indigenous apiculate yeasts has long been viewed as undesirable (Velázquez et al., 1991; Ciani, 1998; Comitini and Ciani, 2010), and methods or factors to limit their proliferation during AF have been described (Farías and Manca de Nadra, 2003; Sosa et al., 2008; Comitini and Ciani, 2010). However, the renewed interest in non-conventional yeasts in the wine industry has led to the reassessment of the species suitable—and beneficial—for winemaking purpose. Several studies report on the characterization of the outcome of AF by *H. uvarum* in mixed or sequential inoculation with *Saccharomyces cerevisiae* in grape must, as *H. uvarum* alone is not able to complete AF (i.e., to consume all the sugar contained in grape musts). Wines resulting from mixed or sequential inoculation of *H. uvarum* and *S. cerevisiae* were shown to differ from pure cultures (*S. cerevisiae*) in their chemical composition. Indeed, the concentrations of some organic acids, aldehydes and minor alcohols (Hong and Park, 2013), higher alcohols and volatile metabolites (Zironi et al., 1993; Zohre and Erten, 2002; Moreira et al., 2011), isoamyl acetate (Moreira et al., 2008), butanediol and acetoin (Romano et al., 1993, 2000), and a few other compounds were reported as significantly different. Some of these alterations could be associated with the secretion of extracellular enzymes. Indeed, several enzymatic activities of technological interest have been characterized, such as β-glucosidase, xylosidase, protease, and lipase activities (Charoenchai et al., 1997; Manzanares et al., 1999; Capece et al., 2005). Moreover, some strains of the *H. uvarum* species were shown to be low producers of ochratoxin A (OTA), the main mycotoxin found in wine (Angioni et al., 2007). For all these reasons, the ability of *H. uvarum* to be preserved by lyophilization and cryopreservation was assessed recently, and was found satisfactory enough to maintain its fermentation ability (de Arruda Moura Pietrowski et al., 2015).

The abiotic and biotic factors to which *H. uvarum* is exposed in grape must have also been investigated. The data showed that the growth of *H. uvarum* was significantly affected by temperature, pH, sulfite, and ethanol concentrations (Gao and Fleet, 1988; Heard and Fleet, 1988; Albertin et al., 2014b), with some of these factors having synergistic or buffering effects. Several authors reported the existence of interactions between *H. uvarum* and *S. cerevisiae* during AF (Mendoza et al., 2007; Wang et al., 2014), associated with various underlying mechanisms including production of killer toxin (Radler et al., 1985, 1990; Schmitt and Neuhausen, 1994), and release of yet unidentified metabolites (Wang et al., 2015).

However, most of these studies evaluated single strains of *H. uvarum*. Only a few authors considered several strains to account for potential diversity within the species (Comi et al., 2001; Capece et al., 2005), but even then, the genetic relationships between the different strains remained obscure due to the lack of dedicated tools. Indeed, the molecular approaches available to date allowed intraspecific discrimination, but not establishment of genetic distance: RAPD (randomly amplified polymorphic DNA, Capece et al., 2005) or restriction endonuclease analysis associated with pulse-field gel electrophoresis (REA-PFGE, Versavaud and Hallet, 1995) were described to discriminate *H. uvarum* strains. By contrast, PCR fingerprinting was not able to discriminate *H. uvarum* strains in Aglianico wines (Caruso et al., 2002). More recently, FT-IR (Fourier transform infrared spectroscopy) was successfully applied to the intraspecific discrimination of *H. uvarum* from grape berries and the winery environment (Grangeteau et al., 2015). However, none of these approaches allows the establishment of genetic relationships between the different isolates. Consequently, the extent of the diversity within the *H. uvarum* species remains uncharacterized.

In this study, 115 strains of *H. uvarum* were isolated from winemaking environments in France and South Africa. Their genetic variability was analyzed using two different approaches: microsatellite markers and AFLP (amplified fragment-length polymorphism). Their phenotypic diversity regarding enzymatic activities and response to environmental factors was also investigated.

## Material and methods

### Yeast strains

One hundred and eleven strains of *Hanseniaspora* spp., including mainly *H. uvarum* and a few *Hanseniaspora guillermondii*, were isolated from French and South African winemaking areas between 2003 and 2014 (Table 1). These strains were identified using molecular techniques like rDNA ITS analysis (Granchi et al., 1999), and sequencing of the D1/D2 domain of 26S rDNA (O'Donnell, 1993; Kurtzman and Robnett, 1998) or the ITS sequence (White et al., 1990; Esteve-Zarzoso et al., 1999). D1/D2 and ITS sequences were then blasted again either NCBI database or YeastIP, a curated yeast database (Weiss et al., 2013).

**Table 1:** *****Hanseniaspora*** sp. strains used in this study**.

**Species**	**Strain**	**Collection^a^**	**Country**	**Year of isolation**	**Substrate**
*H. uvarum*	CRBO L0638	CRBOeno	France	2006	Grape/wine
*H. uvarum*	CRBO L0552	CRBOeno	France	2005	Grape/wine
*H. uvarum*	CRBO L1437	CRBOeno	France	2014	Grape/wine
*H. uvarum*	CRBO L0531	CRBOeno	France	2005	Grape/wine
*H. uvarum*	CRBO L1491	CRBOeno	France	2014	Grape/wine
*H. uvarum*	CRBO L0555	CRBOeno	France	2005	Grape/wine
*H. uvarum*	CRBO L1468	CRBOeno	France	2014	Grape/wine
*H. uvarum*	CRBO L1481	CRBOeno	France	2014	Grape/wine
*H. uvarum*	CRBO L0413	CRBOeno	France	2003	Grape/wine
*H. uvarum*	CRBO L0414	CRBOeno	France	2003	Grape/wine
*H. uvarum*	CRBO L1438	CRBOeno	France	2014	Grape/wine
*H. uvarum*	CRBO L1497	CRBOeno	France	2014	Grape/wine
*H. uvarum*	CRBO L0430	CRBOeno	France	2003	Grape/wine
*H. uvarum*	CRBO L1469	CRBOeno	France	2014	Grape/wine
*H. uvarum*	CRBO L0406	CRBOeno	France	2003	Grape/wine
*H. uvarum*	CRBO L14124	CRBOeno	France	2013	Grape/wine
*H. uvarum*	CRBO L1455	CRBOeno	France	2014	Grape/wine
*H. uvarum*	CRBO L0765	UR Oeno	France	2007	Grape/wine
*H. uvarum*	CRBO L1474	CRBOeno	France	2014	Grape/wine
*H. uvarum*	NZ15	CRPR	New-Zealand	2009	Grape/wine
*H. uvarum*	Gui21	UR Oeno	France	2012	Grape/wine
*H. uvarum*	NZ234	CRPR	New-Zealand	2009	Grape/wine
*H. uvarum*	CRBO L0660	CRBOeno	France	2006	Grape/wine
*H. uvarum*	CRBO L0764	CRBOeno	France	2007	Grape/wine
*H. uvarum*	CRBO L0557	CRBOeno	France	2005	Grape/wine
*H. uvarum*	CRBO L0658	CRBOeno	France	2006	Grape/wine
*H. uvarum*	CRBO L0659	CRBOeno	France	2006	Grape/wine
*H. uvarum*	CRBO L0763	UR Oeno	France	2007	Grape/wine
*H. uvarum*	CRBO L0456	CRBOeno	France	2003	Grape/wine
*H. uvarum*	CRBO L0666	CRBOeno	France	2006	Grape/wine
*H. uvarum*	CRBO L0554	CRBOeno	France	2005	Grape/wine
*H. uvarum*	CRBO L0639	CRBOeno	France	2006	Grape/wine
*H. uvarum*	IWBT Y888	IWBT	South Africa	2011	Grape/wine
*H. uvarum*	CRBO L14118	CRBOeno	France	2014	Grape/wine
*H. uvarum*	CRBO L14150	CRBOeno	France	2014	Grape/wine
*H. uvarum*	CRBO L14144	CRBOeno	France	2014	Grape/wine
*H. uvarum*	CRBO L1442	CRBOeno	France	2014	Grape/wine
*H. uvarum*	NZ5	CRPR	New-Zealand	2009	Grape/wine
*H. uvarum*	CRBO L1433	CRBOeno	France	2014	Grape/wine
*H. uvarum*	CRBO L1492	CRBOeno	France	2014	Grape/wine
*H. uvarum*	CRBO L14112	CRBOeno	France	2014	Grape/wine
*H. uvarum*	CRBO L14136	CRBOeno	France	2014	Grape/wine
*H. uvarum*	Y-1612	NRRL	Indonesia	NA	Soil
*H. uvarum*	Y-915	NRRL	NA	NA	Cider
*H. uvarum*	NZ1	CRPR	New-Zealand	2009	Grape/wine
*H. uvarum*	CRBO L1461	CRBOeno	France	2014	Grape/wine
*H. uvarum*	CRBO L1441	CRBOeno	France	2014	Grape/wine
*H. uvarum*	CRBO L1415	CRBOeno	France	2014	Grape/wine
*H. uvarum*	CRBO L14130	CRBOeno	France	2014	Grape/wine
*H. uvarum*	DSMZ 70285	DSMZ	Germany	NA	Nature
*H. uvarum*	CRBO L14113	CRBOeno	France	2014	Grape/wine
*H. uvarum*	CRBO L0418	CRBOeno	France	2003	Grape/wine
*H. uvarum*	CLIB 303	CLIB	Ukraine	NA	Grape/wine
*H. uvarum*	CRBO L0428	CRBOeno	France	2003	Grape/wine
*H. uvarum*	CRBO L1449	CRBOeno	France	2014	Grape/wine
*H. uvarum*	CRBO L1448	CRBOeno	France	2014	Grape/wine
*H. uvarum*	CRBO L14108	CRBOeno	France	2014	Grape/wine
*H. uvarum*	CRBO L1420	CRBOeno	France	2014	Grape/wine
*H. uvarum*	516149	MAFF (NIAS)	Japan	NA	Nature
*H. uvarum*	CRBO L0401	CRBOeno	France	2003	Grape/wine
*H. uvarum*	CRBO L1462	CRBOeno	France	2014	Grape/wine
*H. uvarum*	CRBO L0665	CRBOeno	France	2006	Grape/wine
*H. uvarum*	CRBO L1414	CRBOeno	France	2014	Grape/wine
*H. uvarum*	CRBO L14149	CRBOeno	France	2014	Grape/wine
*H. uvarum*	CRBO L14143	CRBOeno	France	2014	Grape/wine
*H. uvarum*	CRBO L1404	CRBOeno	France	2014	Grape/wine
*H. uvarum*	CRBO L14129	CRBOeno	France	2014	Grape/wine
*H. uvarum*	CRBO L0312	CRBOeno	France	2003	Grape/wine
*H. uvarum*	Gui1	UR Oeno	France	2012	Grape/wine
*H. uvarum*	IWBT Y1173	IWBT	South Africa	2009	Grape/wine
*H. uvarum*	CRBO L0756	CRBOeno	France	2007	Grape/wine
*H. uvarum*	CRBO L14119	CRBOeno	France	2014	Grape/wine
*H. uvarum*	IWBT Y968	IWBT	South Africa	2014	Grape/wine
*H. uvarum*	TB Sau 1	UR Oeno	France	2012	Grape/wine
*H. uvarum*	IWBT Y952	IWBT	South Africa	2014	Grape/wine
*H. uvarum*	CRBO L1434	CRBOeno	France	2014	Grape/wine
*H. uvarum*	IWBT Y1097	IWBT	South Africa	2009	Grape/wine
*H. uvarum*	CRBO L1446	CRBOeno	France	2014	Grape/wine
*H. uvarum*	CRBO L0743	CRBOeno	France	2007	Grape/wine
*H. uvarum*	CRBO L0744	CRBOeno	France	2007	Grape/wine
*H. uvarum*	IWBT Y864	IWBT	South Africa	2011	Grape/wine
*H. uvarum*	TB Sem 1	UR Oeno	France	2012	Grape/wine
*H. uvarum*	IWBT Y967	IWBT	South Africa	2013	Grape/wine
*H. uvarum*	IWBT Y969	IWBT	South Africa	2014	Grape/wine
*H. uvarum*	CRBO L1427	CRBOeno	France	2014	Grape/wine
*H. uvarum*	CRBO L1486	CRBOeno	France	2014	Grape/wine
*H. uvarum*	IWBT Y1044	IWBT	South Africa	2009	Grape/wine
*H. guilliermondii*	IWBT Y901	IWBT	South Africa	2012	Grape/wine
*H. uvarum*	YB-783	NRRL	USA	NA	Nature
*H. uvarum*	CRBO L1430	CRBOeno	France	2014	Grape/wine
*H. uvarum*	IWBT Y861	IWBT	South Africa	2011	Grape/wine
*H. uvarum*	IWBT Y1116	IWBT	South Africa	2009	Grape/wine
*H. uvarum*	IWBT Y1139	IWBT	South Africa	2009	Grape/wine
*H. uvarum*	CRBO L1487	CRBOeno	France	2014	Grape/wine
*H. uvarum*	CRBO L0551	CRBOeno	France	2005	Grape/wine
*H. uvarum*	CRBO L1445	CRBOeno	France	2014	Grape/wine
*H. uvarum*	Gui3	UR Oeno	France	2012	Grape/wine
*H. uvarum*	CRBO L14125	CRBOeno	France	2014	Grape/wine
*H. uvarum*	Y-1614	NRRL	Russia	NA	Grape/wine
*H. uvarum*	CRBO L1426	CRBOeno	France	2014	Grape/wine
*H. uvarum*	CRBO L0671	CRBOeno	France	2006	Grape/wine
*H. uvarum*	CRBO L14137	CRBOeno	France	2014	Grape/wine
*H. uvarum*	NZ148	CRPR	New-Zealand	2009	Grape/wine
*H. uvarum*	Gui12	UR Oeno	France	2012	Grape/wine
*H. uvarum*	CRBO L1473	CRBOeno	France	2014	Grape/wine
*H. uvarum*	YB-3199	NRRL	USA	NA	Fruit
*H. uvarum*	IWBT Y941	IWBT	South Africa	2013	Grape/wine
*H. uvarum*	Yq NS2	UR Oeno	France	2012	Grape/wine
*H. uvarum*	IWBT Y1100	IWBT	South Africa	2009	Grape/wine
*H. uvarum*	IWBT Y1013	IWBT	South Africa	2009	Grape/wine
*H. uvarum*	IWBT Y1196	IWBT	South Africa	2009	Grape/wine
*H. uvarum*	IWBT Y1177	IWBT	South Africa	2009	Grape/wine
*H. uvarum*	IWBT Y1192	IWBT	South Africa	2009	Grape/wine
*H. uvarum*	IWBT Y1133	IWBT	South Africa	2009	Grape/wine
*H. uvarum*	IWBT Y1190	IWBT	South Africa	2009	Grape/wine
*H. uvarum*	IWBT Y966	IWBT	South Africa	2013	Grape/wine
*H. uvarum*	CRBO L1418	CRBOeno	France	2014	Grape/wine
*H. guilliermondii*	113816	MAFF (NIAS)	NA	NA	Fruit
*H. guilliermondii*	IWBT Y970	IWBT	South Africa	2013	Grape/wine
*H. guilliermondii*	IWBT Y1035	IWBT	South Africa	2009	Grape/wine
*H. guilliermondii*	IWBT Y1165	IWBT	South Africa	2009	Grape/wine
*H. guilliermondii*	Y-1625	NRRL	South Africa	NA	Clinical
*H. opuntiae*	IWBT Y863	IWBT	South Africa	2011	Grape/wine
*H. opuntiae*	IWBT Y875	IWBT	South Africa	2011	Grape/wine
*H. vineae*	IWBT Y907	IWBT	South Africa	2012	Grape/wine
*H. vineae*	IWBT Y971	IWBT	South Africa	2013	Grape/wine

a *CLIB, CIRM-Levures, INRA/AgroParisTech, Thiverval-Grignon, France; CRBOeno, Centre de Ressources Biologiques Œnologie, Villenave d'Ornon, France; CRPR, Centre de Recherche Pernod-Ricard, Creteil, France; DSMZ, Leibniz-Institut DSMZ, Braunschweig, Germany; IWBT, IWBT, Stellenbosch University, South Africa; MAFF (NIAS), NIAS Genebank, Ibaraki, Japan; NRRL, ARS Culture Collection, Peoria, USA; UR Oeno, Research unit Oenology, Villenave d'Ornon, France*.

NA stands for “Not Available.”

Fifteen strains from other geographical and substrate (nature, cider, etc.) origins were included (Table 1). For phenotypic characterization, several control strains were used: *S. cerevisiae* VIN13 (Mocke, 2005) was used as positive control for killer activity, *S. cerevisiae* ZIM 1859 S6 (Zagorc et al., 2001) was used as killer sensitive yeasts. *Metschnikowia pulcherrima* IWBT Y1123, *Schwanniomyces polymorphus* var. *africanus* CBS 8047, *Saccharomyces paradoxus* RO88 (Redzepovic et al., 2003), *Metschnikowia chrysoperlae* IWBT Y955 were used as positive controls for the acid protease, β-glucosidase, pectinase, and chitinase tests, respectively.

All strains were grown at 24°C in traditional YPD medium containing 1% yeast extract, 1% peptone, and 2% glucose (w/v), supplemented or not with 2% agar (w/v).

Microsatellite analysis was applied to all *Hanseniapora* spp. strains available, while only a subset of strains was used for both AFLP and phenotyping assays, more time-consuming and less reproducible over large number of experiments. For AFLP approach, 47 strains were selected, and for phenotyping data we used a subset of 30 strains (all included in the AFLP panel) as well as 10 other *Hanseniaspora* spp.

### Genome sequencing, microsatellite loci identification, and primers design

A draft genomic sequence was produced using Ion Torrent technology. Briefly, a genomic library of strain CRBO L0551 was produced using the Ion Xpress Plus Fragment Library Kit (Life Technologies, Carlsbad, USA), with an enzymatic shearing of 10 min at 37°C. DNA was sequenced on an Ion Torrent PGM (Life Technologies, Carlsbad, CA). After trimming on quality threshold (Phred-type quality score of Q20, QPhred = 20) and length threshold (50 bp) using CLC GenomicsWorkbench 7.0.3 (CLC bio, Boston, MA), Newbler software (version 2.7, 454 Life Sciences) was used to produce a *de novo* assembly of 1665 contigs of more than 1000 bp. This draft assembly forms a 7.68 Mb sequence for an estimated genome size of 8–9 Mb (Esteve-Zarzoso et al., 2001).

Microsatellites (di- to tetranucleotide repeats) were searched within the *de novo* genome assembly as described previously (Albertin et al., 2014a), and primers were designed using the Design primers' tool on the SGD website (http://www.yeastgenome.org/cgi-bin/web-primer) by applying Schuelke's method (Schuelke, 2000) to reduce costs. Amplified fragment sizes varied from 120 to 466 bp, allowing subsequent multiplexing of the amplicons (Table 2).

**Table 2:** **Microsatellite loci for ***Hanseniaspora uvarum*** genotyping**.

**Microsatellite name**	**Motif**	**Fluorescent dye**	**Primers**	**Tm**	**Alleles size range (bp)**	**Coding sequence (blastx result)**	**Ho (observed heterozygosity)**
HU292	TCT	FAM	F: CACGACGTTGTAAAACGACTTCAAATCCGGCATAGTGGT; R: CCATTGTTGCTTCTGAAACC	55	247–253 [2 alleles]	Unnamed protein product in *Kluyveromyces dobzhanskii* CBS 2104 [emb|CDO94325.1]	0.017
HU409	ACA	PET	F: CACGACGTTGTAAAACGACAGATCAAGCAGAGACCACAAA; R: TTGTCGCTTTCTTTGTAAACA	52	429–441 [4 alleles]	Hypothetical protein D499_0Z00400 in *H. uvarum* DSM 2768 [gb|KKA01551.1]	0.087
HU440	ATA	FAM	F: CACGACGTTGTAAAACGACACTGCTGTATTTGCATTTTTG; R: GGAAAAAGCAGAAAAAAAGCG	55	389–451 [9 alleles]	No significant similarity found	0.574
HU467	TGT	NED	F: CACGACGTTGTAAAACGACGGGTTAGCAACGTTTTCGAT; R: TGGATTGGTGAAAATGTGCA	52	267–291 [6 alleles]	No significant similarity found	0.078
HU508	ACA	NED	F: CACGACGTTGTAAAACGACAGGTACAGGCGTTATATCCGA; R: TGGTGTGTGATGTACGTTGTG	55	146–192 [8 alleles]	No significant similarity found	0.07
HU593	AC	PET	F: CACGACGTTGTAAAACGACCTCCTTGCACCCAAAAAAA; R: TTCATCGGCTGTTATGCTTG	52	261–287 [8 alleles]	No significant similarity found	0.2
HU594	AGA	FAM	F: CACGACGTTGTAAAACGACTTTGTCCTTCACTGGTGGTCA; R: TGTTTTGATTCCATTATCGG	52	120–152 [7 alleles]	No significant similarity found	0.33
HU620	TCTT	NED	F: CACGACGTTGTAAAACGACCCTTTGCTGGTAAGGTACCTG; R: GCTTGGAAATGGCTGTTATG	52	428–466 [7 alleles]	Translation initiation factor eIF-2B subunit epsilon in *H. uvarum* DSM 2768 [gb|KKA03659.1]	0.043
HU68	TCT	HEX	F: CACGACGTTGTAAAACGACAAAGGCTATGTTGAGCCTGA; R: AAGGCTTGTTTAAAGCAGGT	52	153–180 [6 alleles]	No significant similarity found	0.096
HU853	TCT	PET	F: CACGACGTTGTAAAACGACGCTTCAGAAATCAAAGCAGC; R: AGGTGCCGGATCTAAATTCA	55	138–166 [5 alleles]	FACT complex subunit HuSPT16 in *H. uvarum* DSM 2768 [gb|KKA03363.1]	0.27

*Allele size in pb. Forward primers were tailed on 5′-end with M13 sequence (CACGACGTTGTAAAACGAC). Tm is the melting temperature used for microsatellite amplification (see Section Materials and Methods)*.

### Microsatellites amplification

DNA was prepared as followed: yeast cells were diluted in 20 mM NaOH (concentration of 1.10^8^ cells/mL), then heated 10 min at 94°C. This solution was used as DNA templates for further PCR reactions.

PCR were performed in a final volume of 15 μL containing 1 μL of DNA template, 0.05 μM of forward primer, 0.5 μM of reverse primer and labeled primer, 1X Taq-&GO (MP Biomedicals, Illkirch, France). Universal M13 primers were labeled with either FAM-, HEX-, PET-, or NED-fluorescent dyes (Eurofins MWG Operon, Les Ulis, France).

Touch-down PCR were carried out using iCycler (Biorad, Hercules, CA) thermal cycler. The program encompassed an initial denaturation step of 1 min at 94°C followed by 10 cycles of 30 s at 94°C, 30 s at Tm + 10°C (followed by a 1°C decrease per cycle until Tm is reached) and 30 s at 72°C, then 20 cycles of 30 s at 94°C, 30 s at Tm and 30 s at 72°C, and a final extension step of 2 min at 72°C.

Amplicons were initially analyzed by a microchip electrophoresis system (MultiNA, Shimadzu) and the optimal conditions for PCR amplifications were assessed. Then, the sizes of the amplified fragments were measured on an ABI3730 DNA analyzer (Applied Biosystems). For that purpose, PCR amplicons were diluted (1800-fold for FAM, 600-fold for HEX, 1200-fold for PET, and 1800-fold for NED-labeled amplicons respectively) and multiplexed in formamide. LIZ 600 molecular marker (ABI GeneScan 600 LIZ Size Standard, Applied Biosystem) was 100-fold diluted and added for each multiplex. Before loading, diluted amplicons were heated 4 min at 94°C. Allele size was recorded using GeneMarker Demo software V2.4.0 (SoftGenetics).

### Microsatellite analysis

Microsatellite analysis, based on allele size, was used to investigate the genetic relationships between isolates. A dendrogram was built using Bruvo's distance (Bruvo et al., 2004) and Ward's clustering, by means of R (R Development Core Team, 2010). Bruvo's distance is particularly well adapted in the case of multiple and/or unknown ploidy levels, which is the case for *H. uvarum* species. Since classical bootstrap resampling is poorly reliable with microsatellite data, we assessed the robustness of the tree nodes using multiscale bootstrap resampling of the loci associated with an approximately unbiased test (Shimodaira, 2002) by means of R and the pvclust package v1.2-2 (Suzuki and Shimodaira, 2006; R Development Core Team, 2010).

Analysis of molecular variance (AMOVA) was performed by means of the pegas package (Paradis, 2010) with *n* = 1000 permutations. We tested whether the genetic distance was significantly explained by geographical localization (i.e., the country of isolation was used as grouping factor) or year of isolation (from 2003 to 2014).

### Amplified fragment length polymorphism

For all yeast species and isolates, genomic DNA was extracted using mechanical cell breakage with glass beads (Hoffman, 2001). DNA concentrations were determined using the NanoDrop™ 1000 spectrophotometer. The AFLP reactions were performed according to Esteve-Zarzoso et al. (2010). Briefly, 1.5 μg DNA was digested for 4 h with *Eco*RI and *Mse*I at 37°C followed by ligation of the *Eco*RI (5′-CTCGTAGACTGCGTACC-3′ and 5′-AATTGGTACGCAGTC-3′) and *Mse*I (5′-GACGATGAGTCCTGAG-3′ and 5′-TACTCAGGACTCAT-3′) adaptors. The primer pair *Eco*RI-0 (5′-GACTGCGTACCAATTC-3′) and *Mse*I-C (5′-GATGAGTCCTGAGTAAC-3′) was used for the non-selective PCR of a 5 μL aliquot of the ligation mix diluted 10 × with TE buffer, while the selective primer was performed using *Eco*RI-C (5′-GACTGCGTACCAATTCC-3′) and *Mse*I-AC (5′-GATGAGTCCTGAGTAAAC-3′) primer pair. The bands were resolved on a 2% (w/v) agarose gel with 1 × TBE buffer at 80 V. The gel was stained with GelRed and visualized under UV. The presence/absence of AFLP markers was scored against a GeneRuler™ 100 bp Plus DNA ladder (Fermentas Life Sciences, Finland) using GeneTools version 4.01 (SynGene, Synoptics Ltd., Cambridge, England). AFLP fragment sizes were rounded to the closest integer and a binary matrix (presence/absence) of 263 AFLP bands, ranging from 94 to 1865 pb was created. A dendrogram was subsequently built using Euclidean's distance, Ward's clustering and multiscale bootstrap resampling.

### Screening for extracellular enzyme activities of enological relevance

All yeast species were grown overnight in YPD broth (Biolab-Merck, Wadeville, South Africa) at 30°C on a rotary wheel. In order to standardize the number of cells spotted, the cultures were diluted to an optical density of 0.1 at a wavelength of 600 nm. On each plate, 10 μL of the diluted culture was spotted and incubated for 3 days at 30°C. The following activities were screened on solid agar media as previously reported in literature.

### β-glucosidase activity

Extracellular β-glucosidase activity was tested on arbutin substrate [1% (w/v) yeast extract, 2% (w/v) peptone, 0.5% (w/v) arbutin, 20 mL 1% ammonium ferric] at pH 3.5 according to the method described by Strauss et al. (2001). *S. polymorphus* var. *africanus* (previously *Debaryomyces polymorphus* var. *africanus*) CBS 8047 was used as a positive control.

### Acid protease activity

This assay was performed according to the method described by Bilinski et al. (1987). Sixty milliliters of phosphate-sodium buffer (24 g/L KH_2_PO_4_ + 35 g/L Na_2_HPO_4_-7H_2_O) was microwaved with 70 mL skim milk solution (100 g/L skim milk in 0.05 M citrate phosphate buffer) for ~45 s or until it starts simmering. Four hundred and eighty milliliters agar (20 g/L, pH adjusted to 3.5) was then added and the plates poured. *M. pulcherrima* IWBT Y1123 (Reid et al., 2012) was used as a positive control for protease activity.

### Polygalacturonase activity

The assay was carried out following the method described by van Wyk et al. (van Wyk and Divol, 2010). Polygalacturonic acid [1.25% (w/v)] was dissolved in 0.68% (w/v) potassium phosphate (pH 3.5), together with 0.67% (w/v) YNB, 1% (w/v) glucose, 2% (w/v) agar. Positive activity was measured against the control yeast *S. paradoxus* RO88 (Mocke, 2005).

### Chitinase activity

Colloidal chitin [0.45% (w/v)] was used as substrate to test for chitinase activity according to the method described by Agrawal and Kotasthane (2012). The pH of the medium was adjusted to 4.7. *M. chrysoperlae* IWBT Y955 was used as a positive control (Ghosh, 2015).

### Screening for killer activity

*Hanseniaspora* spp. isolates were tested for their potential killer activity against *S. cerevisiae* ZIM1859 S6 previously reported as killer sensitive strains. The so-called “spot-on-the-lawn” technique was used, as described by Mehlomakulu et al. (2014). Briefly, all the strains were cultivated overnight in 5 mL YPD broth on a rotary wheel. The cells were harvested by centrifugation and re-suspended in saline [0.9% (w/v) NaCl] to an OD_600nm_ of 0.1 (~3 × 10^7^ cells/mL). To prepare the seeded cultures, 1 mL of the sensitive cells was mixed with 4 mL of a 4% (w/v) pre-autoclaved agar solution and 5 mL of a filter sterilized commercial preservative-free white table grape juice supplemented with 0.5% (w/v) yeast extract, adjusted to pH 4.5. The solution was poured into Petri dishes and allowed to set. Thereafter, 10 μL of overnight cultures of the potential killer strains in saline were spotted on the surface. The plates were incubated at 20°C until a lawn of seeded yeasts was visible and a zone of inhibition around the killer positive strain *S. cerevisiae* VIN13 was observed.

### Sporulation

Sporulation ability of *Hanseniapora* spp. isolates was assessed on three different media: McClary's acetate agar (10% glucose, 1.8 g/L potassium chloride, 8.2 g/L sodium acetate trihydrate, 2.5 g/L yeast extract, 15 g/L agar), malt extract agar (5% malt extract, 2% agar) as described by Kurtzman et al. (2011), and potassium acetate agar (10 g/L Potassium Acetate, 15 g/L agar) by streaking colonies on these media. The cells were then stained according to the method described by Merritt and Hurley (1972).

### Growth assays under various environmental conditions

Strain ability to metabolize glycerol as sole carbon source was tested as followed: strains were plated on 2% glycerol agar plates (1% yeast extract, 2% peptone, 2% glycerol, 1.5% agar) and incubated at 25°C for up to 7 days.

In order to test the impact of low temperature (12°C), anaerobia and the addition of copper solution, yeast strains were grown for 24 h in YNB (BD Difco) pH 3.5 at 25°C with constant agitation then serially 10-fold diluted and spotted on YNB agar plates (pH 3.5). Ten microliters of serial cellular concentrations were tested (10^3^ cells/ml, 10^4^ cells/ml, 10^5^ cells/ml,) and gave similar results. Cellular suspensions were spotted using a Steers multipoint inoculator. Anaerobic conditions were created in sachet by AnaeroGen sachet AN0025 (Oxoid). Actual anaerobia was checked using GasPak™ Dry Anaerobic Indicator Strips (BD). The presence or absence of growth was recorded after 48 h incubation (12°C or 25°C, aerobia or anaerobia).

Susceptibility to copper was estimated by plating the yeast strains on YNB pH 3.5 containing either CuSO_4_ (copper sulfate, the molecule usually contained in Bordeaux mixture) or Cu(OH)_2_ (copper hydroxide, as contained in ChampFlo, Nufarm) at concentrations varying from 0.03 to 32 μg/mL of CuSO_4_ or Cu(OH)_2_ respectively. After 48H incubation at 25°C, the minimum inhibitory concentration (MIC) was determined.

## Results

### Development of microsatellite markers for *Hanseniaspora uvarum*

Next generation sequencing was used to produce a *de novo* assembly of the genome sequence of CRBO L0551, a strain isolated from grape must in Bordeaux region in 2005. Although this *de novo* assembly displayed an important number of contigs (1665 contigs of more than 1000 bp), it was sufficient to locate repeated sequence. Microsatellite loci (dinucleotide to tetranucleotide) were selected on the basis of their location: on different contigs and not within the 5′-end and 3′-end of the contigs (3 kb exclusion in order to exclude possible telomeric or subtelomeric positions). Primers were designed to amplify 11 microsatellite loci, four of them being located within putative coding sequence (Table 2). The amplicons were separated using a microchip electrophoresis system (MultiNA), and the optimal conditions for microsatellites amplifications were assessed on a subpanel of five strains of *H. uvarum* (data not shown). After optimization, the microsatellites markers were tested on other species of the *Hanseniaspora* genus: *H. guillermondii* Y-1625^T^, 113816, IWBT Y1035, IWBT Y1165, IWBT Y901, IWBT Y970; *H. opuntiae* IWBT Y863 and IWBT Y875; *H. vineae* IWBT Y907, and IWBT Y971. No amplification was observed for these non-*uvarum* strains (data not shown), except for strain IWBT Y901. Strain IWBT Y901 was identified as *H. guillermondii* by sequencing both ITS and LSU D1/D2 rRNA regions, yet allowed the normal amplification of all 11 microsatellites markers.

The 11 microsatellites markers were then used to genotype 115 strains, including 101 *H. uvarum* strains isolated from various wineries in France near Bordeaux and in South Africa near Stellenbosch (Table 1). A few other isolates from winemaking environments were added: the type strain Y-1614 from Russia, five strains from New-Zealand (NZ1, NZ5, NZ15, NZ148, and NZ234) and CLIB 303 from Ukraine. Six strains from non-enological environments were also genotyped: Y-1612 (soil, Indonesia), Y-915 (cider), DSMZ 70285 (soil), 516149 (maize, Japan), YB-783 (tree, USA), and YB-3199 (fruit, USA). Strain IWBT Y901, identified as *H. guillermondii* but able to amplify all microsatellites, was also added. All microsatellites were polymorphic on this panel of 115 strains, with only two alleles for HU292 and up to 9 alleles for HU440 (Table 2). Although the polymorphism of the microsatellite loci was limited compared to other species (Legras et al., 2005; Albertin et al., 2014a,c; Masneuf-Pomarede et al., 2015), altogether they were discriminant enough to detect 86 different genotypes over the 115 tested. Twenty strains displayed only one allele per locus, while 95 showed heterozygosity for at least 1 upon 10 loci. Heterozygosity was detected for all loci, with observed heterozygosity ranging from 0.017 for the less polymorphic locus HU292 to 0.574 for HU440, the more polymorphic locus.

### Exploring the genetic relationships between *H. uvarum* isolates using microsatellites

**Figure 1:**
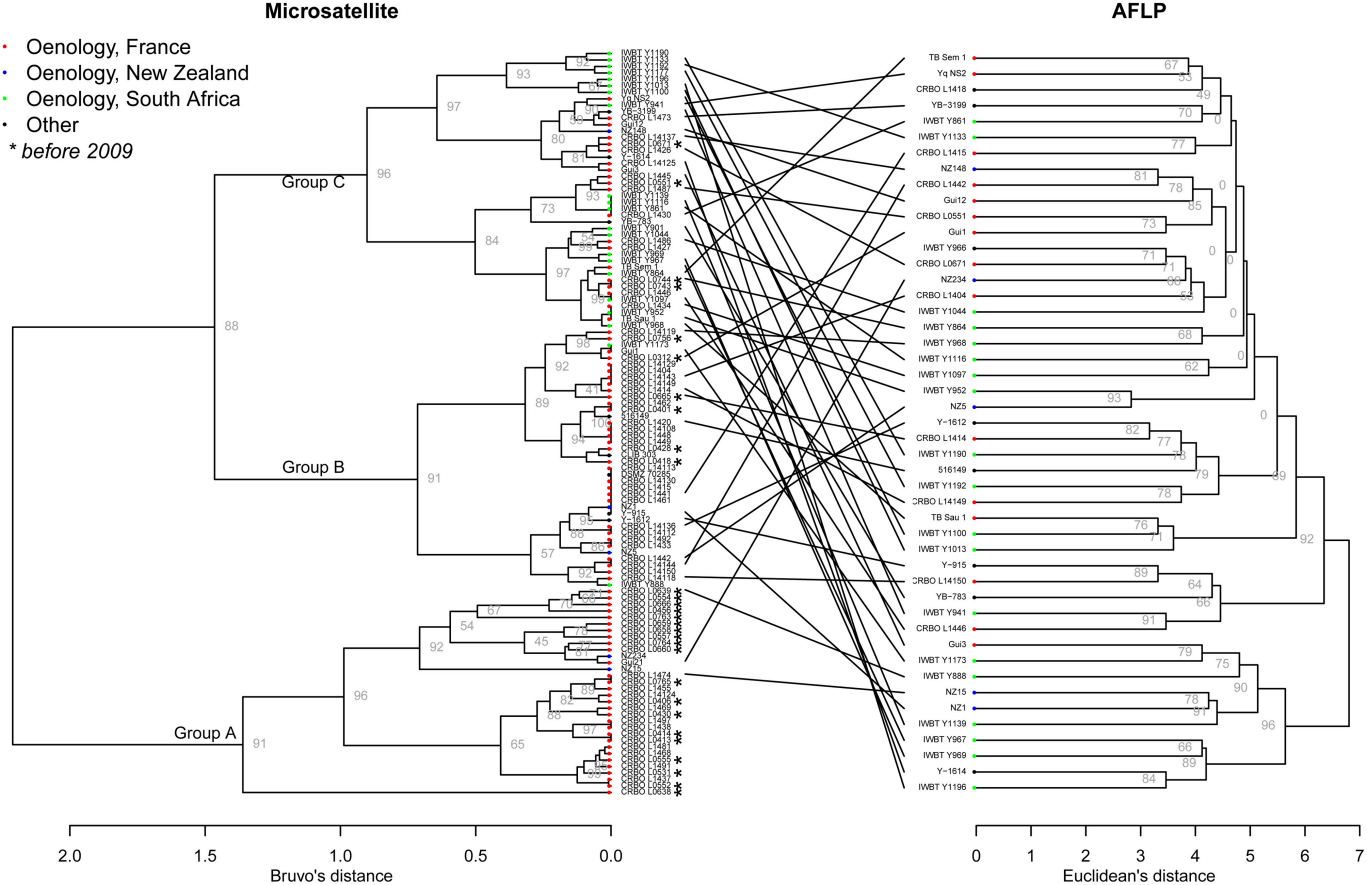
**Dendrogram trees of ***Hanseniaspora uvarum*** from microsatellite dataset (115 strains) and AFLP dataset (47 strains)**. Bruvo'as and Euclidean distance were used for microsatellite and AFLP data, respectively. Ward's clustering and multiscale bootstrap resampling were used in both cases. For visibility, only the boostraps of the higher nodes were shown for microsatellite data.

The genetic relationships between the 115 isolates of *H. uvarum* were studied using Bruvo's distance (Bruvo et al., 2004) and Ward's clustering. The resulting dendrogram (Figure 1) shows three main clusters: one cluster (group C) contained almost all strains from South African winemaking environments (19 of the 21), and was highly supported (bootstrap value of 96). The two other groups contained mostly wine strains from France, but interestingly, these groups clustered on the basis of the year of isolation: most strains collected before 2009 clustered in group A (19 strains upon 29), with high bootstrap value (91). Group B contained 40 strains, most of them (25) being isolated after 2009 from winemaking environments in France (boostrap value of 91).

To confirm the genetic clustering based on both geographical distance and year of isolation, we performed AMOVA. When using the country of origin as grouping factor, AMOVA was significant (*p* = 0.00099), and the country explained 8.54% of the total variation of the microsatellite dataset (Table 3). The year of isolation was also used as a grouping factor, and explained much more variation (20.62%, *p* < 10^−6^). These results confirmed that year of isolation as well as geographical origin significantly shaped the diversity of *H. uvarum* populations from winemaking environments.

**Table 3:** **AMOVA results using microsatellite or AFLP dataset, for country and year of isolation as grouping factors**.

**Dataset**	**Factor**	***p*-value**	**Variation explained by factor**	**Modalities (number of strains)**
Microsatellite	Country	0.00099	8.54%	France (81); South Africa (21); New Zealand (5)
Microsatellite	Year of isolation	<<10^−6^	20.62%	2003 (9); 2005 (6); 2006 (8); 2007 (6); 2009 (17); 2011 (3); 2012 (8); 2013 (3); 2014 (47)
AFLP	Country	0.2258	–	France (16); South Africa (20); New Zealand (5)
AFLP	Year of isolation	0.7323	–	2005 (1); 2006 (1); 2009 (16); 2011 (3); 2012 (6); 2013 (3); 2014 (11)

### Genetic diversity of *H. uvarum* populations in winemaking environments

The wine strains used in this study were isolated from several wineries, sometimes from different samples over several years. This is the case of winery G, for which 19 strains were isolated from grape must between 2003 and 2014 (Table 4). These 19 strains displayed 17 different genotypes distributed throughout the dendrogram, indicating that no clone was a specific signature of this winery. The same pattern was observed for all wineries: in most instances, several genotypes from different genetic groups were identified within the same winery, suggesting that the absence of genetic signature at the winery level was common for *H. uvarum* population.

**Table 4:** **Diversity of ***Hanseniaspora uvarum*** populations for 20 wineries as detected by microsatellite genotyping**.

**Winery**	**Country**	**Number of genotypes/Number of strains**	**Year of isolations**	**Genetic groups**	**Strains ID**
Winery A	France	3 genotypes/4 strains	2007–2009	Groups A-B	CRBO L0413, CRBO L0414, CRBO L0428, CRBO L0430
Winery B	France	3 genotypes/3 strains	2014–2014	Group C	CRBO L1426, CRBO L1427, CRBO L1430
Winery C	France	5 genotypes/6 strains	2003–2014	Groups A-B	CRBO L1414, CRBO L1415, CRBO L1437, CRBO L1438, CRBO L1441, CRBO L1442
Winery D	France	3 genotypes/3 strains	2005–2007	Group B	CRBO L14108, CRBO L14112, CRBO L14113
Winery E	South Africa	2 genotypes/2 strains	2009–2014	Group C	IWBT Y941, IWBT Y967
Winery G	France	17 genotypes/19 strains	2003–2014	Groups A-B-C	CRBO L0312, CRBO L14143, CRBO L14144, CRBO L14149, CRBO L14150, CRBO L1468, CRBO L1469, CRBO L1473, CRBO L1474, CRBO L1481, CRBO L1486, CRBO L1487, CRBO L1491, CRBO L1492, CRBO L1497, Gui1, Gui12, Gui21, Gui3
Winery H	New-Zealand	5 genotypes/5 strains	2005–2011	Groups A-B-C	NZ1, NZ148, NZ15, NZ234, NZ5
Winery I	South Africa	6 genotypes/6 strains	2003–2014	Groups B-C	IWBT Y861, IWBT Y864, IWBT Y888, IWBT Y952, IWBT Y968, IWBT Y969
Winery J	France	4 genotypes/4 strains	2005–2014	Groups B-C	CRBO L14129, CRBO L14130, CRBO L14136, CRBO L14137
Winery L	France	18 genotypes/19 strains	2003–2014	Groups A-B-C	CRBO L0551, CRBO L0552, CRBO L0554, CRBO L0555, CRBO L0557, CRBO L0638, CRBO L0639, CRBO L0658, CRBO L0659, CRBO L0660, CRBO L0665, CRBO L0666, CRBO L0671, CRBO L0743, CRBO L0744, CRBO L0756, CRBO L0763, CRBO L0764, CRBO L0765
Winery M	France	3 genotypes/3 strains	2005–2014	Groups B-C	CRBO L1404, CRBO L1433, CRBO L1434
Winery N	France	1 genotypes/2 strains	2007–2007	Group B	CRBO L1448, CRBO L1449
Winery O	France	2 genotypes/2 strains	2014–2014	Group C	CRBO L1445, CRBO L1446
Winery P	South Africa	3 genotypes/3 strains	2014–2014	Groups B-C	IWBT Y1013, IWBT Y1173, IWBT Y1177
Winery R	South Africa	2 genotypes/2 strains	2012–2012	Group C	IWBT Y1190, IWBT Y1192
Winery S	South Africa	2 genotypes/2 strains	2014–2014	Group C	IWBT Y1116, IWBT Y1196
Winery T	France	6 genotypes/6 strains	2003–2014	Groups A-B-C	CRBO L14118, CRBO L14119, CRBO L14124, CRBO L14125, TB Sau 1, TB Sem 1
Winery U	South Africa	2 genotypes/2 strains	2012–2014	Group C	IWBT Y1097, IWBT Y1100
Winery X	France	2 genotypes/2 strains	2009–2014	Groups A-B	CRBO L0401, CRBO L0406
Winery Y	France	4 genotypes/4 strains	2006–2014	Groups A-B-C	CRBO L1455, CRBO L1461, CRBO L1462, Yq NS2

We also studied the genetic diversity at sample level. For example, five strains from grape must were isolated and genotyped from the same sample in winery. The five strains (CRBO L0743, CRBO L0756, CRBO L0763, CRBO L0764, and CRBO L0765) exhibited five different genotypes distributed on the tree. In addition, in 2005, for winery L, some strains were isolated on days 1, 3, 4, and 5 during the pre-fermentative stage of the same tank. The four corresponding strains (CRBO L0552, CRBO L0554, CRBO L0555, and CRBO L0557) clustered in group A on the dendrogram, but they all displayed different genotypes. Altogether, these results suggest that the diversity of the population of *H. uvarum* is high in winemaking environments and that no specific genetic signature exists in a given winery.

### Comparing microsatellite and AFLP typing

AFLP techniques are viewed as moderately repeatable over a high number of experiments, so that AFLP analyses are usually applied to a limited number of strains in order to be reliable. Here, we chose to apply AFLP analysis to a subpanel of 47 strains, in order to compare the results obtained from microsatellite data and AFLP data. The AFLP dendrogram was produced using Euclidean distance and Ward's clustering (Figure 1). Comparison between microsatellite and AFLP dendrograms revealed important differences with no obvious clustering for AFLP data for country origin or vintage, even when comparing exactly the same subset of strains (Supplementary Figure 1). We subsequently performed AMOVA analysis using the distance matrix produced from AFLP data (Table 3). When using the country or the year of isolation as grouping factor, AMOVA was not significant (*p* > 0.05) using AFLP data, indicating that AFLP clustering was not able to detect the genetic structure depending on geographical origin nor year of isolation. However, it has to be noted that AFLP tool was able to discriminate 47 strains upon 47 on the basis of their AFLP patterns. By contrast, on the same subset, the microsatellite tool identified 37 different genotypes. This indicated that although AFLP tool lacked robustness to assess the genetic relationship of individuals, it was more discriminant than the microsatellite tool.

### Phenotyping *Hanseniaspora* sp. isolates

As phenotyping assays are time-consuming, a subpanel of 30 strains of *H. uvarum* and 10 other *Hanseniaspora* spp. were selected and subjected to various plate assays to assess whether they possessed any extracellular enzyme activity that could be of interest in enology (Table 5). Their killer activity against two strains of *S. cerevisiae* that are sensitive to *S. cerevisiae*'s killer toxins was also investigated. Finally, their ability to grow when exposed to various environmental factors of scientific or enological interest (low temperature, anaerobia, copper presence, glycerol as the only carbon source) was recorded.

**Table 5:** **Phenotyping 45 ***Hanseniaspora*** sp. strains for enzymatic activities and growth ability**.

**Substrate**	**Activity**					**Growth**				
	**β-glucosidase**	**Protease**	**Pectinase**	**Pectinase**	**Chitinase**	**Glycerol**	**12°C**	**Anaerobia**	**CMI CuSO _4_(μg/mL)**	**CMI Cu(OH)_2_ (μg/mL)**
	**Arbutin**	**Skim milk**	**PG Agar**	**PG Agarose**	**Chitin**	**–**	**–**	**–**	**–**	**–**
516149	LB	1	0	–	–	w	+	+	4	4
NZ1	LB	1	0	–	–	–	NA	NA	NA	NA
NZ148	LB	0	–	–	–	w	+	+	4	2
NZ15	LB	1	–	–	–	w	+	+	2	2
NZ234	LB	0	w	–	1	G	+	+	2	4
NZ5	LB	1	0	–	–	–	+	+	4	4
TB Sau 1	LB	1	–	–	–	w	+	+	4	2
TB Sem 1	LB	1	0	–	–	w	+	+	4	2
IWBT Y1013	LB	1	–	–	–	w	NA	NA	NA	NA
IWBT Y1044	LB	1	–	–	–	w	NA	NA	NA	NA
IWBT Y1097	LB	1	–	–	–	w	NA	NA	NA	NA
IWBT Y1100	LB	–	–	–	–	w	+	+	2	4
IWBT Y1116	G	–	–	–	–	w	+	+	2	4
IWBT Y1133	LB	1	–	–	–	w	NA	NA	NA	NA
IWBT Y1139	LB	–	–	–	–	w	NA	NA	NA	NA
IWBT Y1173	LB	–	–	–	–	w	+	+	2	2
IWBT Y1177	LB	–	–	–	–	w	+	+	2	2
IWBT Y1190	LB	–	–	–	–	w	+	+	4	4
IWBT Y1192	LB	–	–	–	–	w	NA	NA	NA	NA
IWBT Y1196	LB	–	–	–	–	w	NA	NA	NA	NA
Y-1612	LB	1	–	–	–	w	+	+	2	2
Y-1614	LB	1	0	–	–	w	–	–	2	2
IWBT Y861	LB	1	–	–	1	w	+	+	4	2
IWBT Y864	LB	1	–	–	–	w	+	+	2	4
IWBT Y888	LB	–	–	–	–	w	+	+	2	4
Y-915	LB	1	0	–	–	w	+	+	4	2
IWBT Y941	LB	0	–	–	–	w	NA	NA	NA	NA
IWBT Y952	LB	0	–	–	–	w	+	+	2	4
IWBT Y966	LB	1	–	–	–	w	NA	NA	NA	NA
IWBT Y967	LB	1	–	–	–	w	NA	NA	NA	NA
IWBT Y968	LB	1	–	–	–	w	NA	NA	NA	NA
IWBT Y969	LB	–	–	–	–	w	NA	NA	NA	NA
YB-3199	LB	1	0	–	–	w	+	+	2	2
YB-783	LB	4	0	–	–	w	+	+	2	4
Yq NS2	LB	0	0	–	–	w	+	+	4	4
**Other** ***Hanseniaspora*** **sp**.										
*H. guilliermondii* 113816	LB	1	–	–	–	w	+	+	1	2
*H. guilliermondii* IWBT Y901	G	–	–	–	–	w	NA	NA	NA	NA
*H. guilliermondii* IWBT Y970	G	–	–	–	–	w	NA	NA	NA	NA
*H. guilliermondii* IWBT Y1035	LB	1	–	–	–	w	NA	NA	NA	NA
*H. guilliermondii* IWBT Y1165	LB	–	–	–	–	w	NA	NA	NA	NA
*H. guilliermondii* Y-1625	LB	1	0	–	–	w	–	+	2	2
*H. opuntiae* IWBT Y863	LB	0	–	–	–	w	NA	NA	NA	NA
*H. opuntiae* IWBT Y875	LB	–	–	–	–	w	NA	NA	NA	NA
*H. vineae* IWBT Y907	LB	–	–	–	–	w	NA	NA	NA	NA
*H. vineae* IWBT Y971	LB	–	–	–	–	w	NA	NA	NA	NA
**CONTROLS**										
*S. polymorphus* CBS 8047	12	0	G	–	6	G	NA	NA	NA	NA
*S. paradoxus* RO88	G	0	12	W	–	G	NA	NA	NA	NA
*M. pulcherrima* IWBT Y1123	2	10	G	–	W	G	+	+	4	2
*M. pulcherrima* IWBT Y1072	2	11	G	–	W	G	NA	NA	NA	NA
*S. cerevisiae* VIN13	G	–	W	–	–	G	NA	NA	NA	NA
*M. chrysoperlae* IWBT Y955	2	W	G	–	1	G	NA	NA	NA	NA

*Zone sizes are indicated in mm, after subtracting the colony size from the total diameter of the zone; + or − indicates growth or absence of growth under these conditions; NA stands for “Not Available”; G indicates that there was colony growth but no extracellular enzyme activity was observed; W indicates very weak growth on the respective plate (single colonies could be observed within the spotted zone); LB indicates a color change in the colony, but no halo was observed*.

With regards to extracellular enzyme activity, all strains showed growth on the arbutin plates, although no distinct halo could be observed. Most *Hanseniaspora* strains showed a slight browning of the colony which might be due to weak activity or even intracellular β-glucosidase activity. Weak acid protease activity was observed for most strains, with YB-783 showing the largest halo (4 mm) and strains Gui21 and CRBO L0551 showing halos of 2 mm after 72-h incubation. No polygalacturonase activity was observed. Finally, weak chitinase activity could be visualized in isolates NZ234 and Y-861 (1-mm halo) while the other isolates showed no growth on the chitin media.

None of the strains was able to sporulate. Regarding their growth ability, most strains were unable to grow on 2% glycerol agar plates after incubation at 30°C for up to 7 days. Their growth ability under various environmental conditions was tested: ability to grow at low temperature (12 and 30°C), under oxic or anoxic, in presence of various copper concentrations. The MIC for copper sulfate and copper hydroxide was either 2 or 4 mg/L with no specific correlation between these 2 factors. All strains tested showed similar ability to grow under these conditions of enological interest, and limited phenotypic variations were recorded. Indeed, only strain Y-1614 did not show any growth at 12°C and anaerobiosis. Finally, no killer activity was observed against *S. cerevisiae*.

## Discussion

### Comparing microsatellite and AFLP genotyping

In this paper, we compared the intraspecific clustering using two different techniques: AFLP and microsatellites. Both approaches allowed discrimination at the strain level: 47 different patterns were scored for AFLP (for 47 strains), while 86 genotypes were evidenced for 115 strains with microsatellite data. Indeed, both methods proved to be discriminant as previously reported (Mariette et al., 2001; Gaudeul et al., 2004), with AFLP having a higher discriminant power in our case. However, it has to be noted that using AFLP, the amplification of multiple bands in a single run may lead to competition between amplicons and therefore to differences of band intensity that complicate data analysis. In addition, AFLP techniques are usually viewed as moderately repeatable thereby making the technique usually poorly reliable, while the repeatability of microsatellites markers is usually higher (Jones et al., 1997) and can be thus applied to a larger number of individuals.

Moreover, AFLP markers are non-codominant markers, so that homozygosity or heterozygosity is difficult to assess (Gaudeul et al., 2004). By contrast, microsatellites are codominant markers, allowing assessing heterozygosity status. Here, we found that 95 out of 115 strains showed heterozygosity, allowing an unprecedented insight into the genetics of the species. Microsatellites are widely used to estimate relatedness among individuals or differentiation among groups. By contrast, AFLP should be taken with caution due to the lack of complete genotypic information caused by dominance (Parker et al., 1998). Indeed, the dendrogram obtained by both approaches are not comparable and the genetic structure based on year of isolation and geographical origin evidenced using microsatellite was completely missed by AFLP analysis.

As expected, microsatellite genotyping proved to be a better tool for establishing genetic relation between strains and getting new insights within species at genetic level (Ross et al., 1999). By contrast, AFLP is interesting to perform assays where genetic relatedness is not needed, which is usually the case for several biotechnological applications in enology: assessing the global population diversity, testing for the prevalence/implantation of a specific (known) strain, searching for contamination evidence, etc. In these latter instances, the technical simplicity and rapidity of AFLP, associated with low cost, is definitively advantageous compared to microsatellite genotyping.

### New insights into the genetic structure of *Hanseniaspora uvarum* from winemaking environments

Like many non-conventional yeasts of enological interest, the genetic structure of *H. uvarum* from winemaking environments remained elusive. Here, using microsatellite data, we show that an important number of *H. uvarum* strains (95/115) are heterozygous. This result could be congruent with the hypothesis of a diploid species, although the possibility of aneuploidy could not be excluded. Additional work should be performed to confirm its diploid status, but will be complicated by the absence of sporulation on classical medium. The absence of sporulation could be explained, at least in part, by the weak ability of the species to metabolize glycerol, suggesting poor respiration ability (sporulation being strongly linked to respiration ability in *S. cerevisiae*, Codon et al., 1995). Indeed, all *H. uvarum* strains (except Y1614) showed unperturbed growth under anaerobic conditions, indicating that respiratory metabolism is not necessary for their normal growth.

Interestingly, strain IWBT Y901 –showing 456/456 identities for D1/D2 sequence with *Hanseniaspora guilliermondii* CBS 465^T^- was clustered among *H. uvarum* strains in group C. While some microsatellite markers can cross the species, most microsatellites are intraspecific. The fact that IWBT Y901 is the sole *H. guillermondii* strain to be amplified by all 11 markers is clearly unusual. The possibility of a contamination of this strain with an actual *H. uvarum* strain can be eliminated as IWBT Y901 differed from all other strains we genotyped: its closest relative IWBT Y1044 differs from two alleles at two different loci. One possible explanation for these unexpected results is that IWBT Y901 could derived from an interspecific hybrid between *H. uvarum* and *H. guillermondii*, a hypothesis that remains to be demonstrated formally.

Microsatellite analysis also reveals a genetic structure related to geographical localization. Since we genotyped mostly strains from France and South Africa, it could be interesting to extend our analysis to strains from other countries in order to assess the extent of relationship between genetic structure and geographical origin. A few wine yeasts were shown to be genetically structured, at least partially, by geography, as it is the case of *Saccharomyces uvarum, Candida zemplinina*, or *Torulaspora delbrueckii* (Albertin et al., 2014a; Almeida et al., 2014; Masneuf-Pomarede et al., 2015). More surprisingly, our data show a strong relationship between the year of isolation and the genetic structure. This result indicates that *H. uvarum* populations isolated from winemaking environments show a temporal clustering in addition to a spatial one. More data are required to determine whether this temporal variation exists only for French and South African strains over the period we tested (2003–2014), or if this trend is also detected for other vineyards and/or larger periods of time. In addition, further investigation is required to determine which factor(s) could be related to this temporal evolution. Factors to be tested include environmental factors such as temperature, pH, sugar concentrations, ability to survive from season to season, etc. Viticultural and enological practices should also be considered, including phytosanitary treatments, sulfite addition, cold pre-fermentation stage, turbidity, or starter culture addition that were shown to impact *Hanseniaspora* populations during the early stage of AF (Albertin et al., 2014b).

Finally, our data failed to identify any specific genetic signature associated with wineries and/or samples. Moreover, we globally identified high level of genetic diversity within all wineries/samples tested, with no evidence for clonal dominance. Such high genetic diversity was previously shown for *Hanseniaspora* populations in grape must and other environments of the winery using FT-IR (Grangeteau et al., 2015). High diversity was also detected for the wine yeast *C. zemplinina* (Masneuf-Pomarede et al., 2015), while other wine species like the spoilage yeast *Brettanomyces bruxellensis* showed clonal populations and maintenance over decades in winemaking environments (Albertin et al., 2014c). *H. uvarum* is known to be insect associated and therefore we can speculate that its diversity may depend on the diversity and frequency of insect occurrence during ripening (Lam and Howell, 2015). Such hypothesis could be tested by investigating the insect-associated yeast diversity and comparing it with the grape/winery diversity.

### *H. uvarum* displays low phenotypic variability for the traits investigated in this study

In order to investigate whether the genetic clustering evidenced above reflects a certain phenotypic diversity, a number of phenotypes of enological relevance were tested: secretion of typical enzymes, as well as ability to grow at cold temperature (similar to that occurring at the beginning of the winemaking process and possibly during the fermentation of white wine), in anaerobiosis (typically occurring during wine fermentation) and ability to tolerate copper, a typical anti-fungal treatment used in the vineyard. The ability to grow on glycerol as sole source of carbon was also tested, following a preliminary observation that *H. uvarum* could not utilize glycerol (not shown). Although both microsatellites and AFLP revealed large genetic variation, the phenotypic variability was very low for the factors investigated (Table 4). Indeed, with a few exceptions, most strains exhibited similar extracellular enzyme activity, tolerance to copper and ability to grow at low temperature or poor ability to use glycerol as sole source of carbon. No clear connection could be observed between these phenotypes and the genetic clustering reported above. *H. uvarum* is usually categorized as a good producer of extracellular enzymes (Dizy and Bisson, 2000) and it is typically reported to exhibit all the enzymatic activities investigated in this study, although this seems to be strain dependent. However, most authors did not adjust the pH of their screening media to wine pH. There seems to be a general consensus between our data and previous studies, that when pH is adjusted to 3.5, most strains of *H. uvarum* display β-glucosidase and protease activities (Lagace and Bisson, 1990; Charoenchai et al., 1997), but not polygalacturonase activity (Charoenchai et al., 1997). No study however investigated the actual impact of these extracellular enzymes on wine composition. All the strains investigated in our study except one could grow at 12°C. This is in agreement with literature. Indeed, it has been reported that low temperatures favor biomass production in *H. uvarum* (Ciani et al., 2006; Mendoza et al., 2009). Surprisingly, *H. uvarum* seems to be poorly able to consume glycerol, even in the presence of oxygen. None of the strains investigated in this study were found able to sporulate. They should therefore all be classified as *H. uvarum* (teleomorph) and not *K. apiculata* (anamorph). As reviewed by Jolly et al. (2006), the region of isolation seems to play a role in the distribution of *H. uvarum* and *K. apiculata*. In temperate regions, an equal mixture of teleomorph/anamorph is found, while in warmer climates, only the teleomorph *H. uvarum* is detected. Yet our strains were isolated from both temperate and warm climate regions (France and South Africa, respectively) and none of the strains studied here were found able to sporulate. Another explanation could be the amount of time that these strains spent as freeze cultures. Indeed, some authors have reported that the time between isolation and analysis plays a role in the ability of *H. uvarum* to sporulate (see review by Jolly et al., 2006).

Overall, with regard to the traits investigated in this study, *H. uvarum* seems to display very little phenotypic variability. In literature, a greater diversity seems to occur in terms of intracellular metabolism. Indeed, several studies report on the influence of *H. uvarum* inoculated in pure or mixed culture with *S. cerevisiae* and describe its production of esters, higher alcohols, and fatty acids (Moreira et al., 2008; Suzzi et al., 2012). These studies are not always in full agreement, as mentioned by the latter authors, and these would point out toward some intraspecific diversity at this level. Nevertheless, our results show that upon inoculation in grape juice, *H. uvarum* could survive the typical cold temperatures applied early in the winemaking process and during the fermentation of white wine as well as the anaerobic conditions also occurring during fermentation. Furthermore, it could potentially release glycosylated compounds and break down proteins through the activity of its extracellular enzymes, both properties being of strong enological interest. Since *H. uvarum* displayed no killer activity against the strains of *S. cerevisiae* tested, these two yeast species could be co-inoculated without threatening the overall proceedings of AF.

In conclusion, we describe in this paper a new analytical tool (microsatellite markers) that allowed estimating the genetic diversity and the genetic relationship between *H. uvarum* from winemaking environments. Our results indicate that *H. uvarum* populations are structured by both geographical origin and the year of isolation from a genetic viewpoint. By contrast, the phenotypic variability was more limited regarding extracellular enzymatic activities and response to environmental factors. Subsequent analysis of a larger number of isolates will help determine the extent of such results in winemaking environments.

## Author contributions

WA and MS designed and performed most of the experiments. CM and TM performed the screening experiments. BC, JC, PG, and VM performed microsatellite development. MP and FS performed genome sequencing and microsatellite analysis. WA, MME, MB, BD and IMF conceived the project, wrote and edited the manuscript.

### Conflict of interest statement

The authors declare that the research was conducted in the absence of any commercial or financial relationships that could be construed as a potential conflict of interest.

## Supplementary material

Supplementary Figure 1 **Comparaison of dendrogram trees of *Hanseniaspora uvarum* obtained from microsatellite and AFLP approaches on the same subset of strains**. Bruvo'as and Euclidean distance were used for microsatellite and AFLP data, respectively.
