# Supplementary figures and images for: Hanseniaspora uvarum from Winemaking Environments Show Spatial and Temporal Genetic Clustering
Source: Front Microbiol. 2016 Jan 20;6:1569. doi: 10.3389/fmicb.2015.01569 (PMC4718985; doi:10.3389/fmicb.2015.01569)

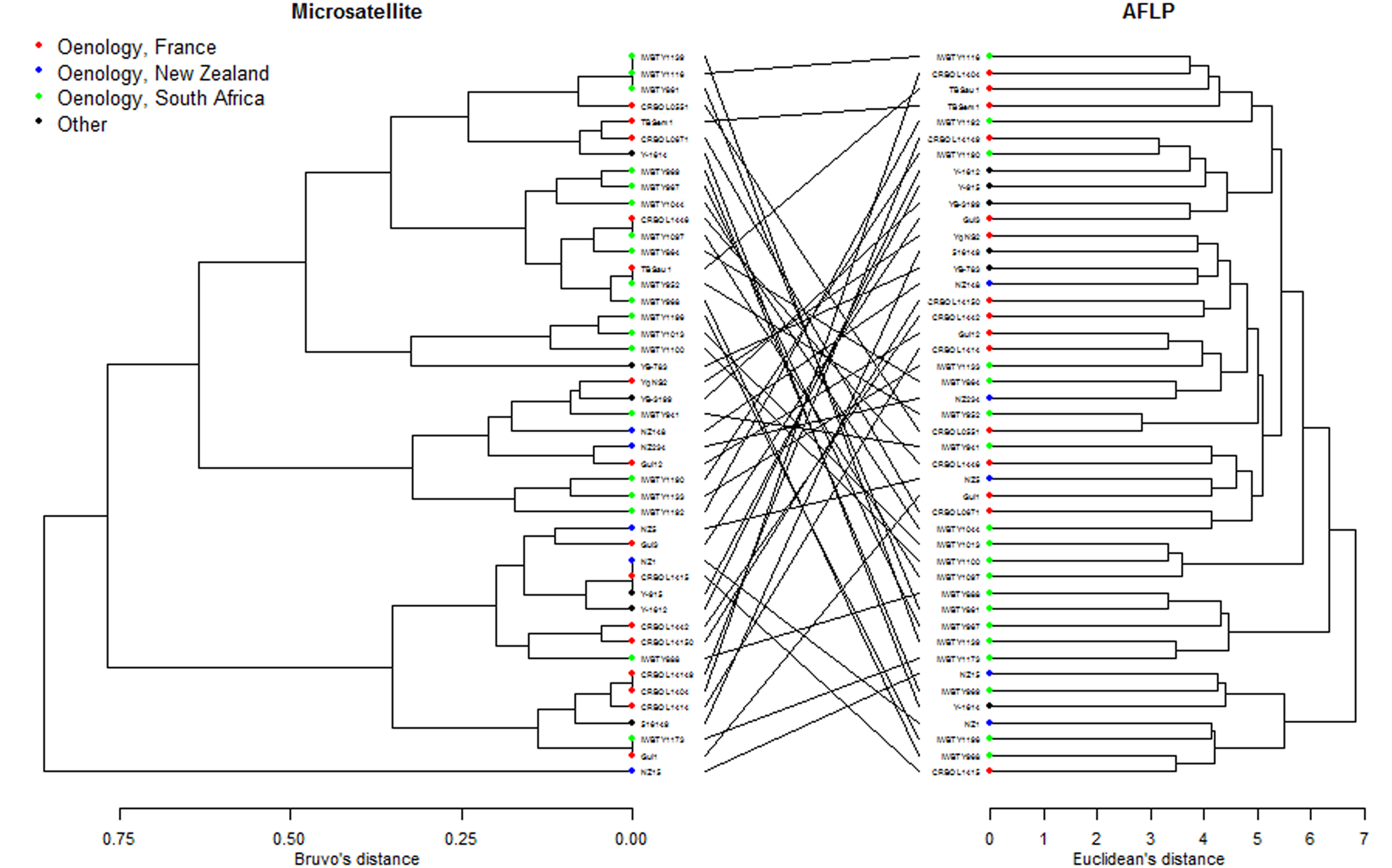

Supplement: Supplementary Figure 1 — Comparaison of dendrogram trees of Hanseniaspora uvarum obtained from microsatellite and AFLP approaches on the same subset of strains. Bruvo'as and Euclidean distance were used for microsatellite and AFLP data, respectively. [file Image1.TIFF]
